# Supplementary material for: RAF inhibitors activate the integrated stress response by direct activation of GCN2
Source: Nat Commun. 2025 Nov 17;16:10033. doi: 10.1038/s41467-025-65376-w (PMC12623982; doi:10.1038/s41467-025-65376-w)
Supplement: Supplementary file 1 — Supplementary Information [file 41467_2025_65376_MOESM1_ESM.pdf]

Supplementary Figure legends

Supplementary Figure 1. RAF inhibitors inhibit cell proliferation independent of paradoxical ERK1/2 activation. a & b. NCI-H358 cells were treated with the indicated concentrations of Dabrafenib (blue circle), Vemurafenib (green diamond), PLX-72840 (dark green circle) , Encorafenib (dark brown triangle), GDC-0879 (brown circle) or Trametinib (black diamond) for 24 hours with a pulse of 10µM EdU for the final hour. Cells were fixed and permeabilized for EdU detection, immunofluorescence with a p-ERK1/2 antibody and co-stained with DAPI. Mean signal per cell was determined by high-content image analysis of 2000-15000 cells per condition. Normalised mean values ± SEM are shown, n = 3 biological replicate experiments analysed for p-ERK1/2 (**a**) or intensity of EdU staining (**b**). **c & d.** NCI-H358 cells were treated with 30 (red triangle), 60 (green diamond) or 100nM (dark blue triangle) Selumetinib for 1 hour prior to addition of the indicated concentration of Dabrafenib for 24 hours. Some cells received increasing concentrations of Selumetinib (dark blue triangle) or Trametinib (black triangle) alone for 24 hours. p-ERK1/2 (**c**) and intensity of EdU staining (**d**) were quantified by HCM analysis as above. Results shown are mean ± SEM of n=2 biological replicate experiments.

Supplementary Figure 2. RAF inhibitors drive the rapid inhibition of DNA replication but do not activate the DNA damage response. a. NCI-H358 cells were treated with 1µM Dabrafenib (blue), 10µM Vemurafenib (bright green), 100nM Trametinib (orange) or 10µM Etoposide (green) for 24hrs with a pulse of EdU for the final hour. Cells were harvested and EdU intensity of cells in S phase was measured by flow cytometry. Top panel showing gating strategy. Cells were gated on forward scatter versus side scatter area (FSCA v SSCA) to remove debris then further gated on forward or side scatter height versus area (FSCH v FSCA and SSCH v SSCA) to gate for single cell populations. This gate was then analysed for EdU intensity v DAPI to separate the phases of cell cycle. S phase cells were plotted for EdU intensity under the indicated treatments. **b.** NCI-H358 cells treated with 1µM Dabrafenib or

10μM Etoposide for 24 hours were fixed and permeabilized for immunofluorescence with p-H2AX (Ser139) antibody, co-stained with DAPI and analysed by confocal microscopy. **c & d.** NCI-H358 cells were treated with 1μM Dabrafenib (blue square), 1μM PLX-7284 (red triangle) or 10μM Etoposide (green triangle) for up to 24 hours. Cells were then fixed and permeabilized for immunofluorescence with (**c**) p-H2AX(S139) or (**d**) p-CHK1(S345) antibodies. Mean signal per cell was determined by high-content image analysis of 2000-15000 cells per condition, n = 1.

Supplementary Figure 3. RNA-seq reveals that Dabrafenib activates the unfolded protein response independently of MEK1/2-ERK1/2 signalling. NCI-H358 cells were treated with control (DMSO), 1μM Dabrafenib (Dab), 30nM Selumetinib (Sel) or the combination (Dab+Sel) for 4hrs or 24hrs, with 4 dishes of cells for each condition/timepoint. Samples were processed for RNA-seq as described in the Results and Methods. **a.** RNA-seq data for the 'ERK1/2 target genes' *DUSP4, DUSP5* and *DUSP6* displaying Log2 counts per million mapped reads for each sample, colour-coded by treatment group with symbols representing 4 hours (circles) or 24 hours (diamonds). **b.** RNA-seq data was mapped to the Hallmark genesets. The camera function from the limma package was used to test whether each gene set was highly ranked relative to other genes. The heatmap shows genesets below FDR of 0.01 with tiles colored by significance level (signed, -log10(p.val)). The Unfolded Protein Response geneset was induced by both Dab and Dab+Sel but not Sel alone. **c**. RNA-seq data for ten 'UPR genes' displaying Log2 counts per million mapped reads for each sample, colour-coded by treatment group with symbols representing 4 hours (circles) or 24 hours (diamonds). All UPR genes were induced by Dab and Dab+Sel but largely unaffected by Sel alone.

Supplementary Figure 4. RAFi and ER stressors drive rapid, strong expression of ATF4 but RAFi drive very weak expression of other ER stress markers such as CHOP and

XBP-1. NCI-H358 cells were treated with either 1μM Dabrafenib (blue square) , 1 μM PLX-7284 (red triangle), 100nM Thapsigargin (green triangle) or 2μg/ml Tunicamycin (black diamond) for up to 24 hours. Cells were then fixed and permeabilized for immunofluorescence with antibodies to ATF-4 (**a**) CHOP (**b)** or XBP-1 (**c**). Mean signal per cell was determined by high-content image analysis of 2000-15000 cells per condition.

Supplementary Figure 5. RAF inhibitors, but not ER stressors, require GCN2 kinase activity to activate the ISR. a. NCI-H358 cells were treated with DMSO, 300nM ISRIB or 300nM A-92 for 30 mins prior to the addition of either 1μM Dabrafenib (Dab), 1μM PLX7284 (PLX), 10μM Vemurafenib (Vem), 3μM Encorafenib (Enc), 1μM LY-3009120 (LY), 3μM PLX8394 (8394) or 100nM Thapsigargin (Tg) for 4 hours. Whole cell lysates were fractionated and immunoblots were probed with the indicated antibodies and captured by LiCor. Results shown are from a single experiment representative of 3 independent experiments. The ratio of p-GCN2 to total GCN2 was quantified from blots and normalised to WT DMSO control. Relative values ± SEM are shown from n = 3 biological replicate experiments. (DMSO = grey bars, ISRIB = pink bar and A-92 = turquoise bar). Dab v DMSO, **p* = 0.0472 as determined by one sample (two tailed) t test. Dab -/+ A92i, **p* = 0.0421 as determined by unpaired t test with Welch’s correction (refer to source data file). **b. & c.** NCI-H358 cells were treated with the indicated concentrations of either ISRIB (**b**) or A-92 (**c**) for 30 mins prior to the addition of DMSO, 100nM Thapsigargin (Thaps’, purple triangle) or 2μg/ml Tunicamycin (Tun’, red circle). Cells were treated for 7 hours with a pulse of 10μM EdU for the final hour, then fixed and permeabilized for EdU detection, immunofluorescence with ATF4- or CHOP-specific antibodies and co-stained with DAPI. Mean signal per cell was quantified by high-content image analysis of 2000-15000 cells per condition. Normalised mean values ± SD are shown, from n = 3 biological replicate experiments (refer to source data file).

Supplementary Figure 6. Knockdown or knockout of GCN2 blocks RAFi-induced activation of the ISR. a. NCI-H358 cells were either mock transfected or transfected with 10nM of siRNA either non targeting (siN-T) or targeted to GCN2 (siGCN2) or ATF4 (siATF4) for 48 hours cells before treatment with DMSO or 1µM Dabrafenib (Dab) for 4 hours. Whole cell lysates were separated by SDS-PAGE and immunoblots were probed with the indicated antibodies. Blots from a single representative experiment are shown. The ratio of p-GCN2 to total GCN2 and ATF4 to tubulin was quantified from blots and normalised to WT DMSO control. Relative values ± SEM are shown from n = 3 biological replicate experiments. (DMSO= grey bar, siNT= pink bar, siGCN2 = turquoise bar and siATF4 = purple bar). Dab treatment with or without siGCN2, *p = 0.0472 as determined by unpaired t test and Welch's correction (see source data file).

Supplementary Figure 7. The majority of RAF inhibitors drive paradoxical activation of both ERK1/2 and the ISR. NCI-H358 cells were treated with either (**a & b**) group 1 RAF inhibitors (Dabrafenib (blue circle), PLX-7284 (red triangle), Vemurafenib (green diamond), Encorafenib (purple triangle), or GDC-0879 (brown circle)) or (**c & d**) group 2 and paradox breaker RAFi (LY-3009120 (blue circle), AZ-628 (red triangle), PLX-8394 (green diamond) or PLX-7922 (purple triangle)) for 8 hours. Cells were then fixed and permeabilized for immunofluorescence with antibodies to p-ERK1/2 (**a & c**) or ATF-4 (**b & d**). Mean signal per cell was determined by high-content image analysis of 2000-15000 cells per condition and normalised to DMSO control. Normalised mean values ± SEM are shown, n = 2-4 (see source data file).

Supplementary Figure 8. Knockdown of CRAF inhibits Dabrafenib-induced activation of ERK1/2 but not activation of GCN2 and the ISR. a. NCI-H358 cells were either mock transfected (MOCK) or transfected with 10nM of siRNA either non targeting (siNT), targeted to ARAF (siARAF) , BRAF (siBRAF), CRAF (siCRAF) or a combination of all (siCombo). After

48 hours cells were treated with either DMSO or 1μM Dabrafenib for 4 hours and whole cell lysates fractionated by SDS-PAGE and immunoblotted with the indicated antibodies. **b-e.** NCI-H358 were transfected as above and after 48 hours were treated with either DMSO, 1μM Dabrafenib, 1μM PLX7284, 10μM Vemurafenib, 3μM Encorafenib, 1μM LY-3009120, 3μM PLX8394, 100nM Thapsigargin or 2μg/ml Tunicamycin for 7 hours. Cells were then fixed and permeabilized for immunofluorescence with antibodies to **b.** BRAF **c.** CRAF **d.** p-ERK1/2 or **e.** ATF4 and co-stained with DAPI. Mean signal per cell was determined by high-content image analysis of 2000-15000 cells per condition. Normalised mean values ± SEM are shown, n = 3 biological replicate experiments.

Supplementary Figure 9. RAF inhibitors activate the integrated stress response by direct activation of GCN2 dimers. The ISR protein kinase GCN2 is an obligate dimer that interacts with ribosomes where it is activated by uncharged deacylated tRNAs and/or the ribosomal P-stalk complex following amino acid starvation[41]. Activated GCN2 then phosphorylates eIF2α to inhibit 5' Cap-dependent mRNA translation, whilst allowing the translation of select mRNAs with an unusual 5' UTRs such as the ATF4 bZip transcription factor which drives a homeostatic gene expression programme allowing cells to adapt to stress. Here we show that RAF inhibitors (RAFi), activate GCN2 leading to eIF2α phosphorylation. This inhibits the expression of key cell cycle regulators (e.g., CDC25A), thereby inhibiting DNA replication. RAFi also drive the expression of ATF4 and the ISR which is prevented by the GCN2 kinase inhibitor A-92, GCN2 RNAi, GCN2 knock-out or ISRIB (an eIF2α antagonist). Activation of the ISR by RAFi was abolished by a GCN2 kinase dead mutation whilst a M802A GCN2 gatekeeper mutant was activated at lower RAFi concentrations, demonstrating that RAFi bind directly to the GCN2 kinase domain; this was supported independently by HDX-MS analysis[57]. RAFi activated full-length recombinant GCN2 dimers *in vitro* and in cells, generating a characteristic 'bell-shaped' concentration-response curve, reminiscent of RAFi-driven paradoxical activation of WT RAF dimers. This

suggests a similar mechanism of activation in which RAFi bind to one protomer of GCN2 and inhibit it, but in doing so drive allosteric activation of the drug-free, ATP-bound dimer partner through the interactions at the dimer interface; this was supported by mechanistic structural models of RAFi interaction with GCN2. Since the ISR is a critical pathway for determining cell survival or death, our observations may be relevant to the clinical use of RAFi, where paradoxical GCN2 activation is a previously unappreciated off-target effect that may modulate tumour cell responses.

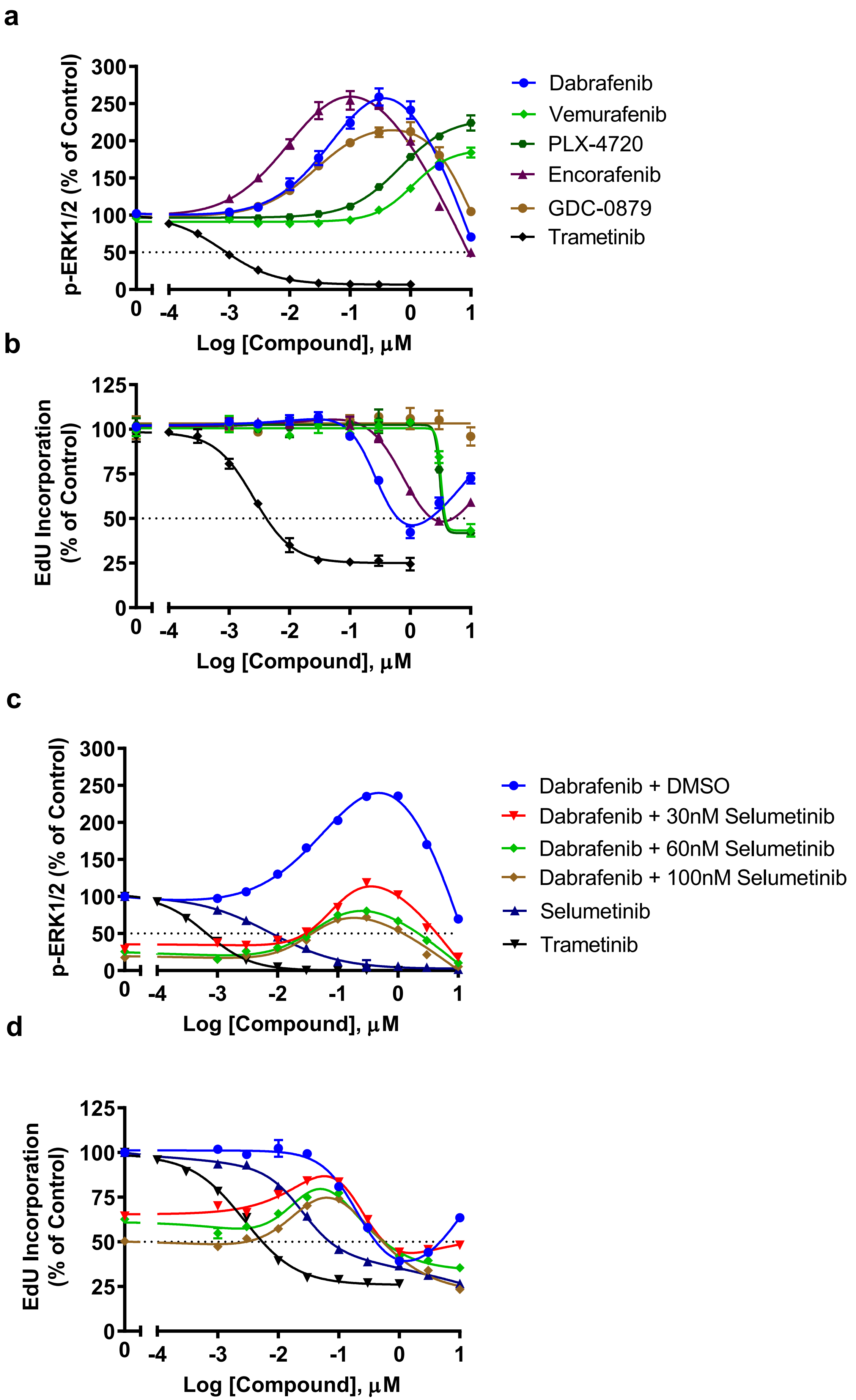
a
p-ERK1/2 (% of Control)
300
250
200
150
100
50
0
0
-4
-3
-2
-1
0
1
Log [Compound], μM
Dabrafenib
Vemurafenib
PLX-4720
Encorafenib
GDC-0879
Trametinib
b
EdU Incorporation
(% of Control)
125
100
75
50
25
0
0
-4
-3
-2
-1
0
1
Log [Compound], μM
c
p-ERK1/2 (% of Control)
300
250
200
150
100
50
0
0
-4
-3
-2
-1
0
1
Log [Compound], μM
Dabrafenib + DMSO
Dabrafenib + 30nM Selumetinib
Dabrafenib + 60nM Selumetinib
Dabrafenib + 100nM Selumetinib
Selumetinib
Trametinib
d
EdU Incorporation
(% of Control)
125
100
75
50
25
0
0
-4
-3
-2
-1
0
1
Log [Compound], μM

Supplementary Figure 2

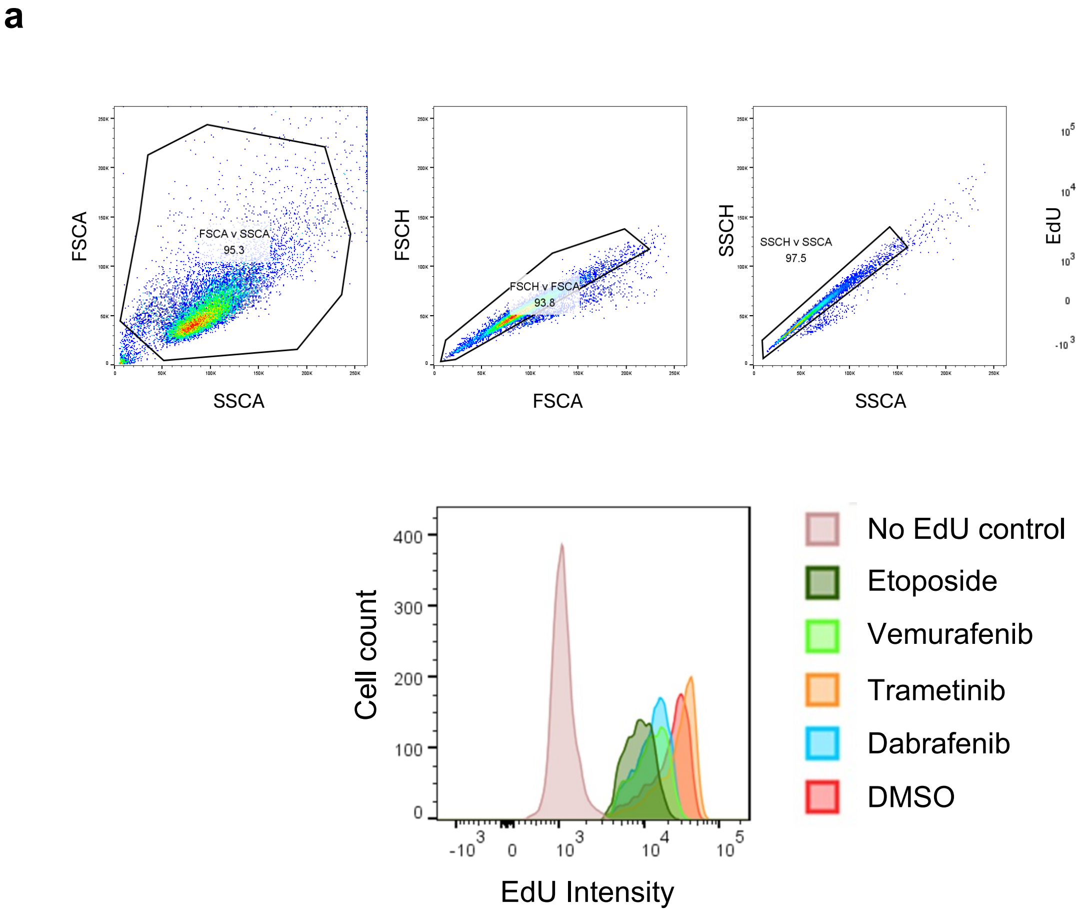

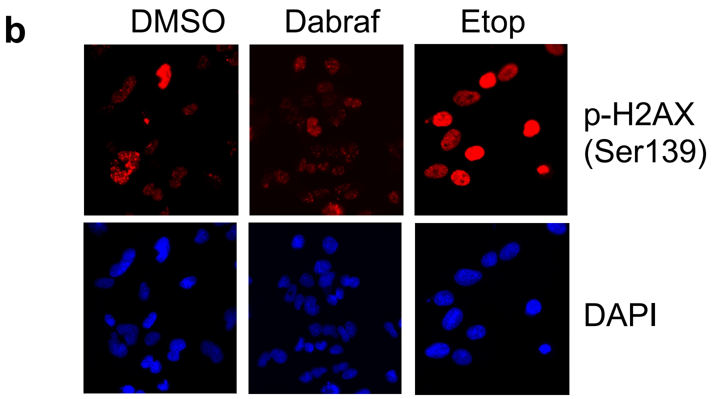

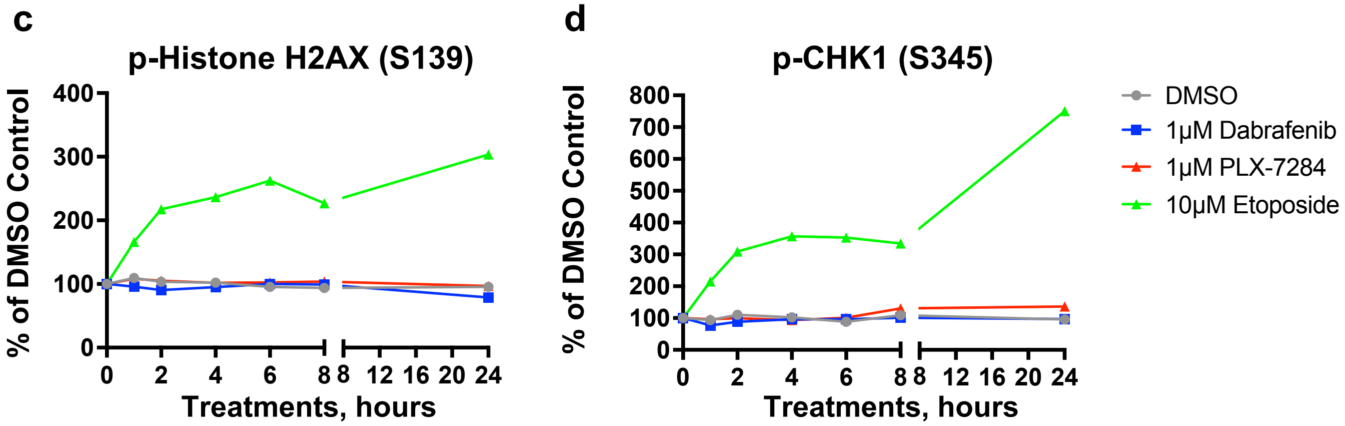

Supplementary Figure 3

a

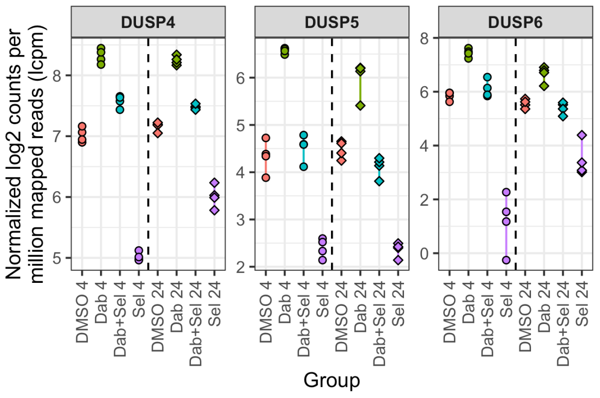

b

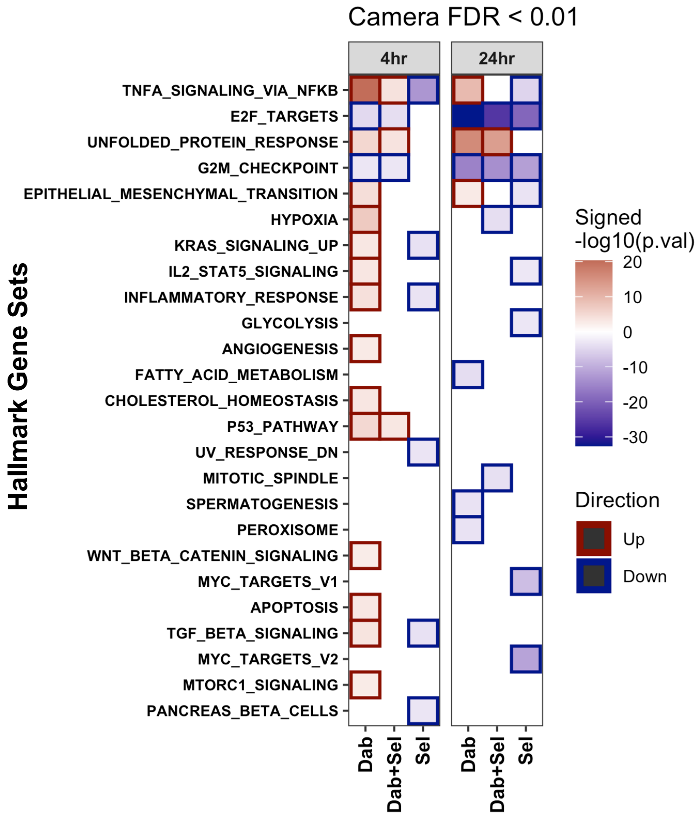

c

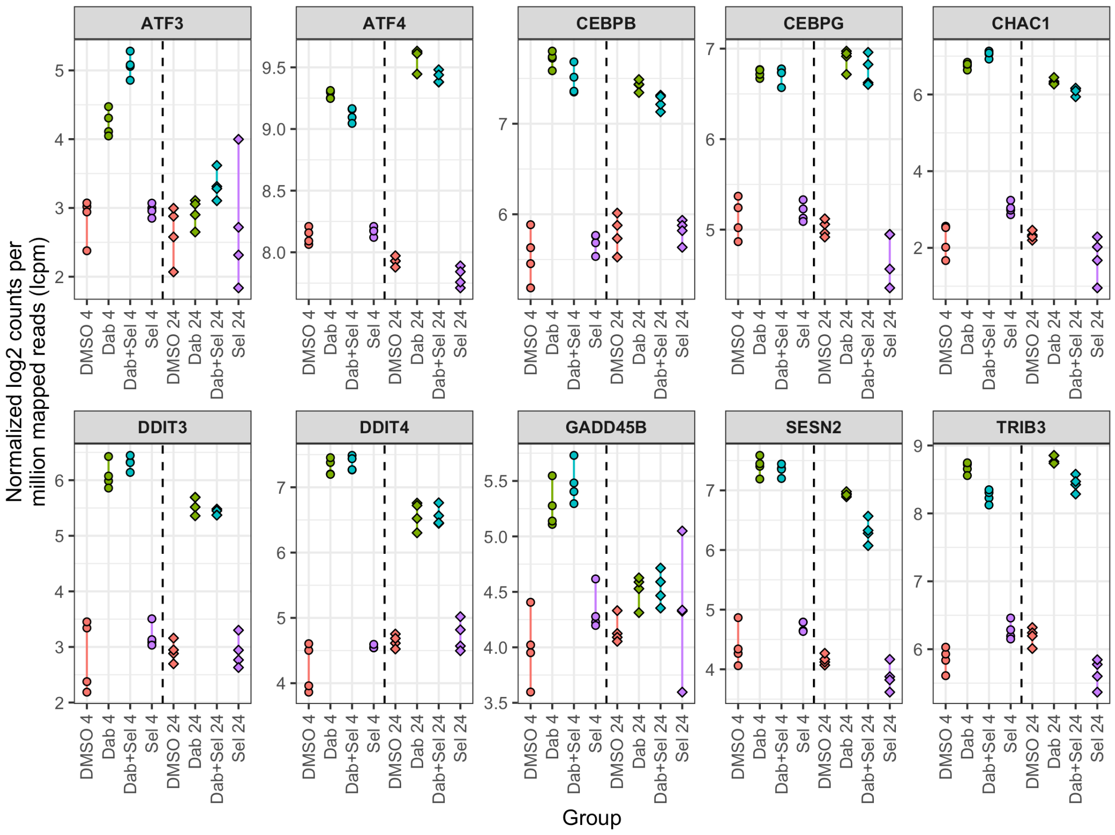

Supplementary Figure 4

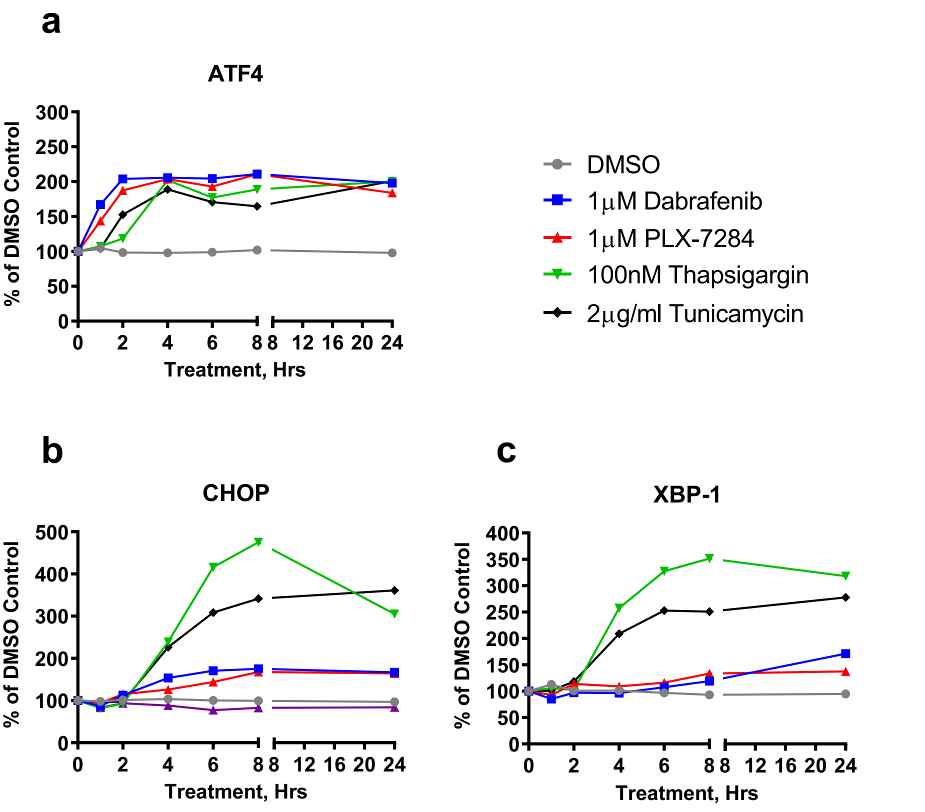

Supplementary Figure 5

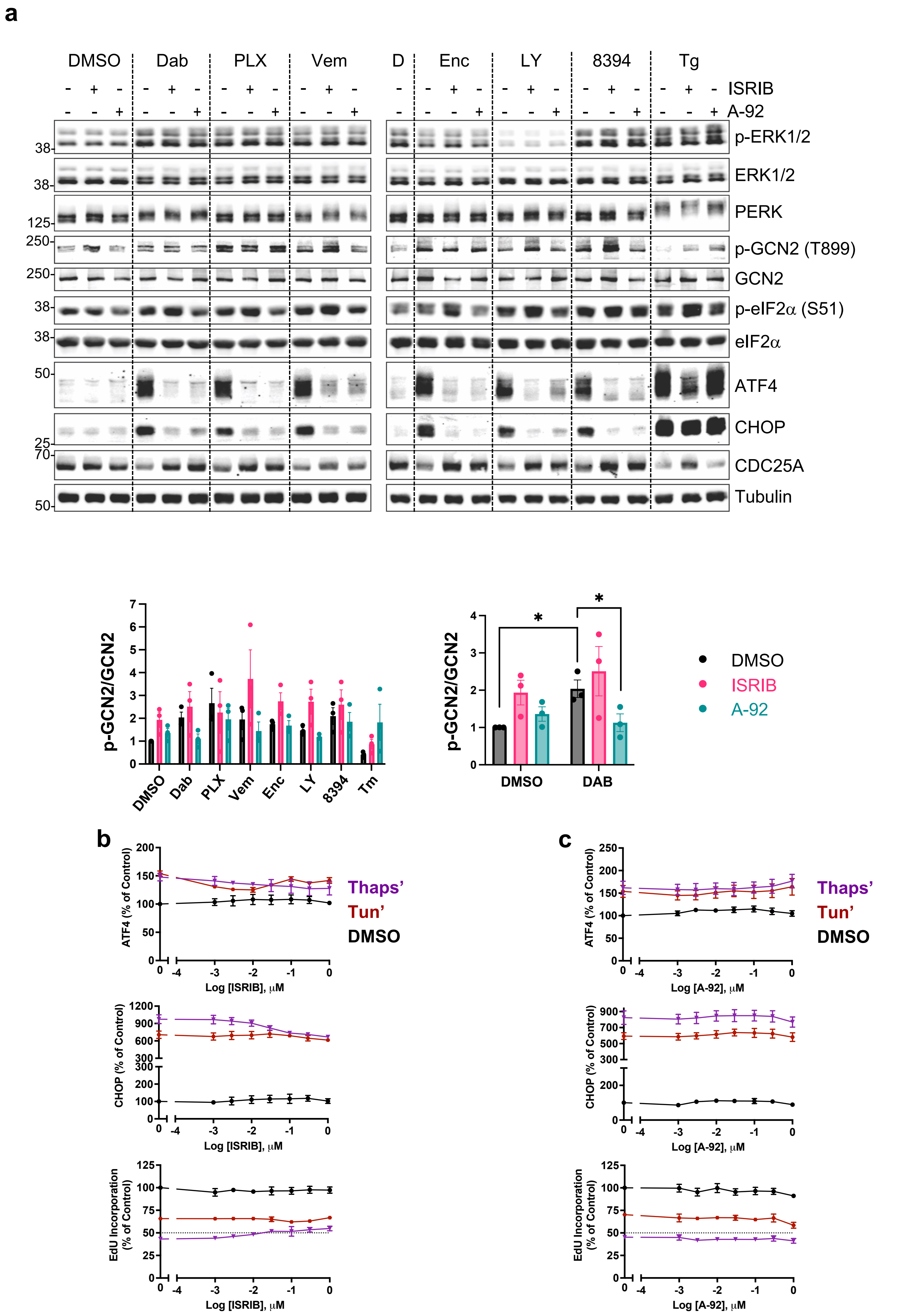

Supplementary Figure 6

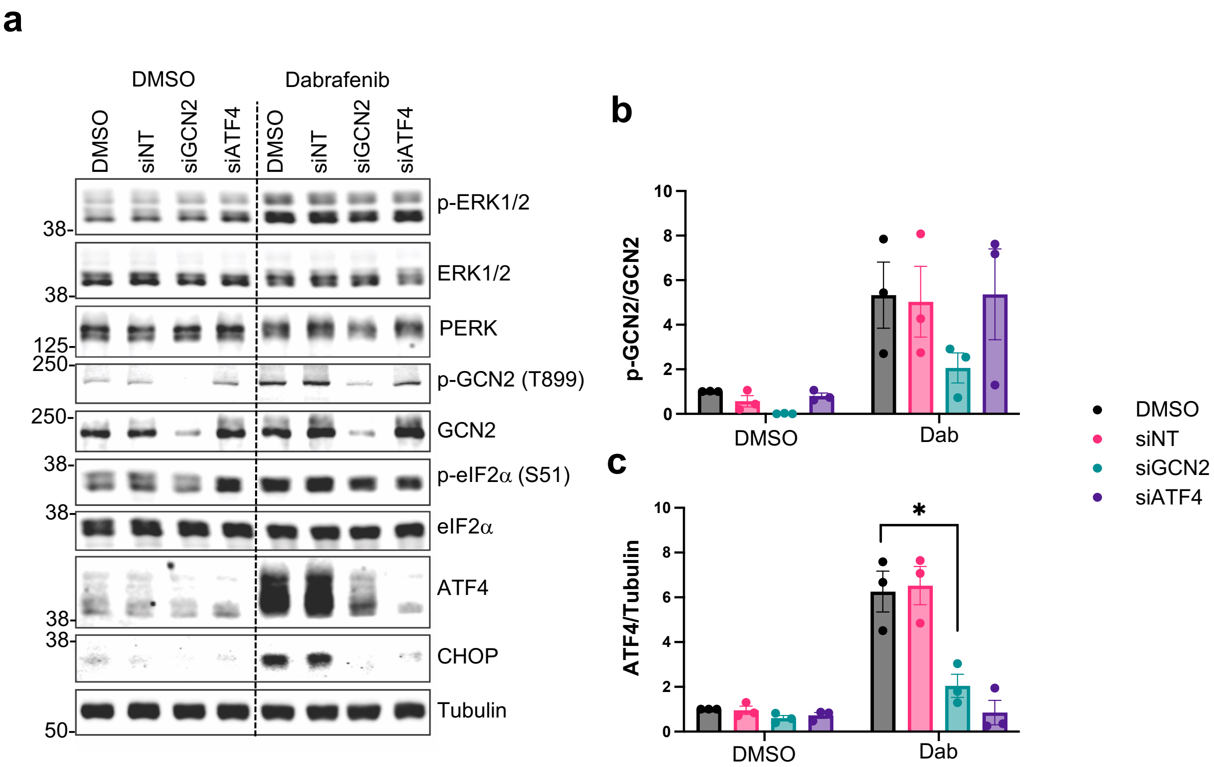

Supplementary Figure 7

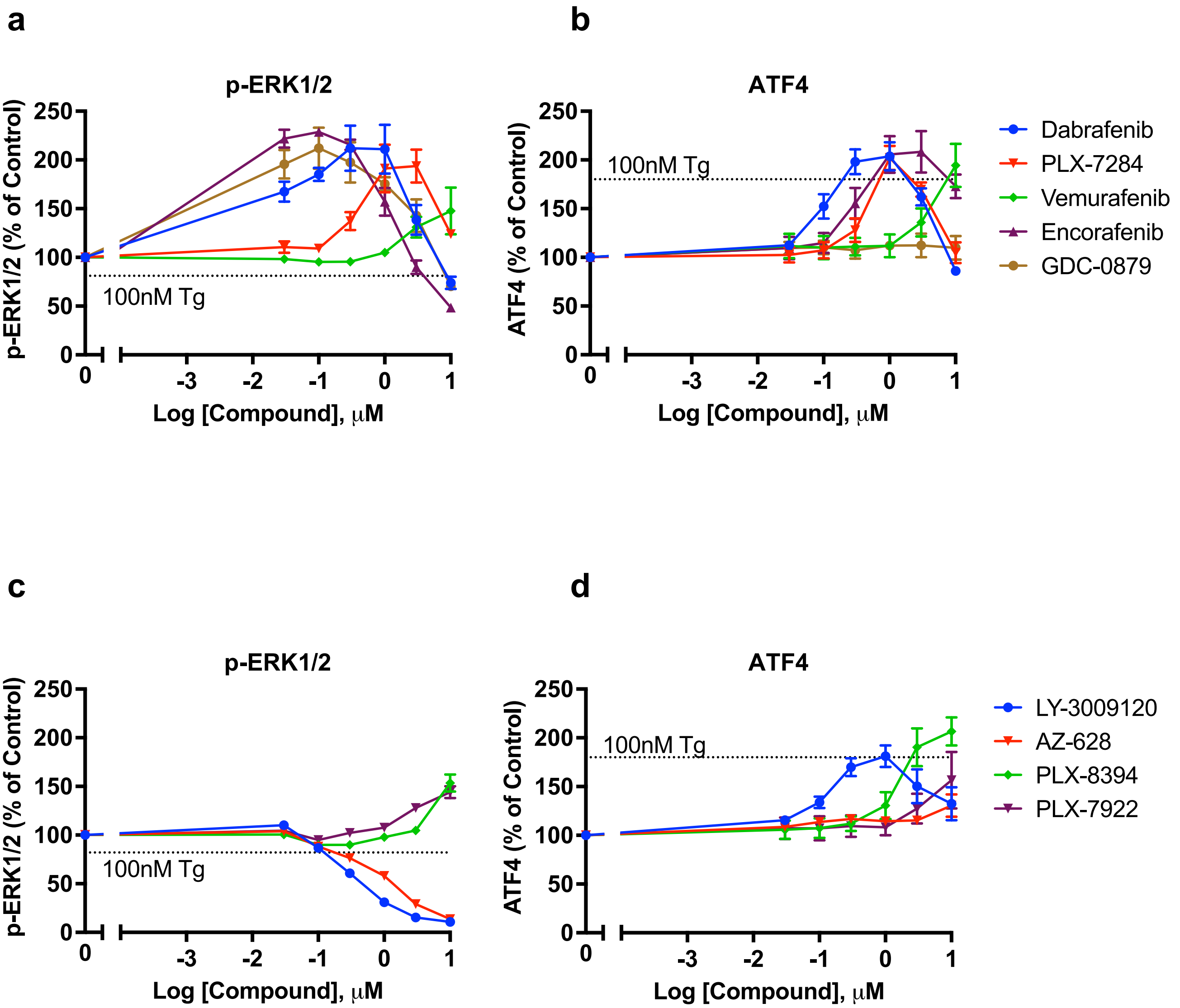

Supplementary Figure 8

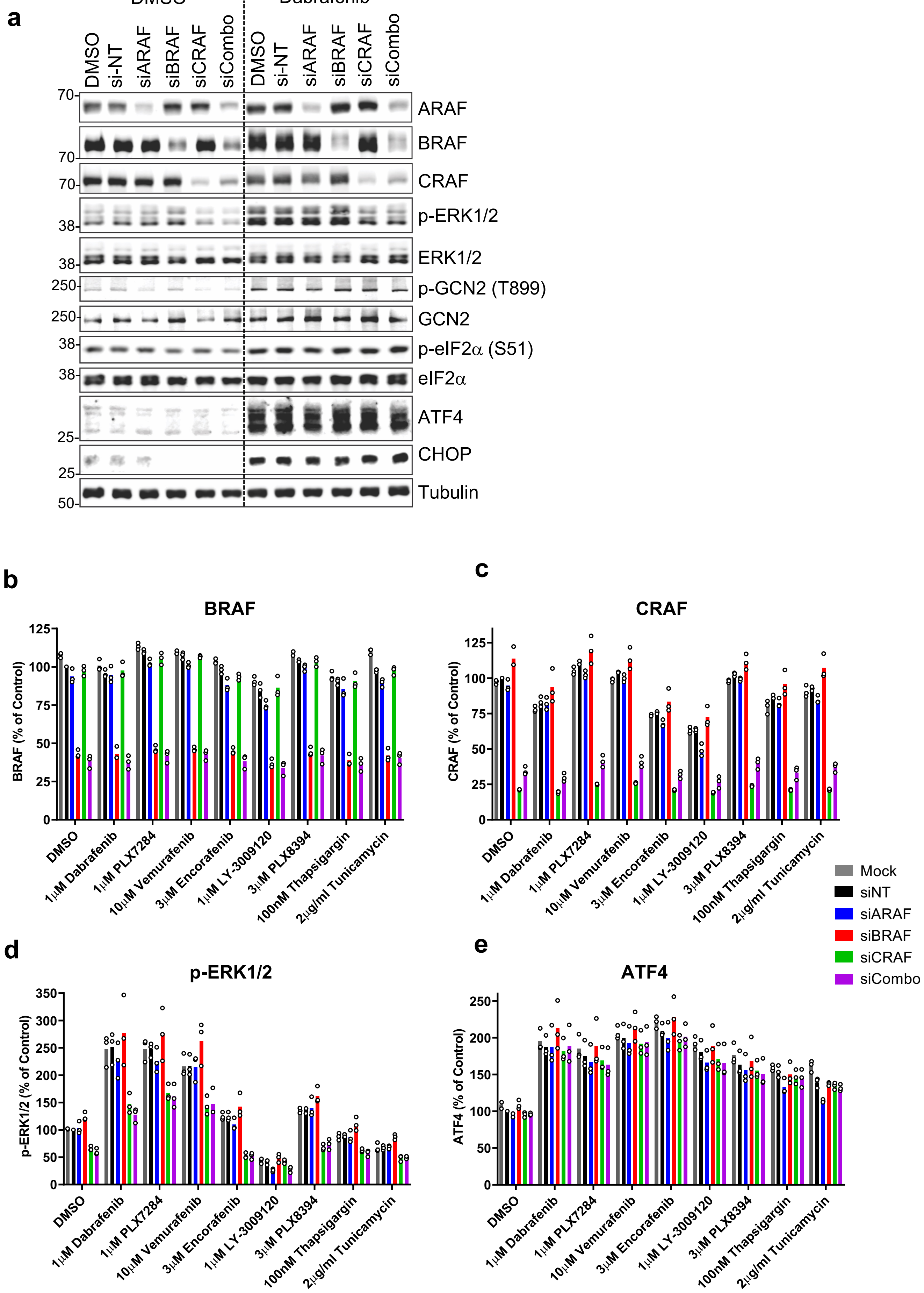

Supplementary Figure 9

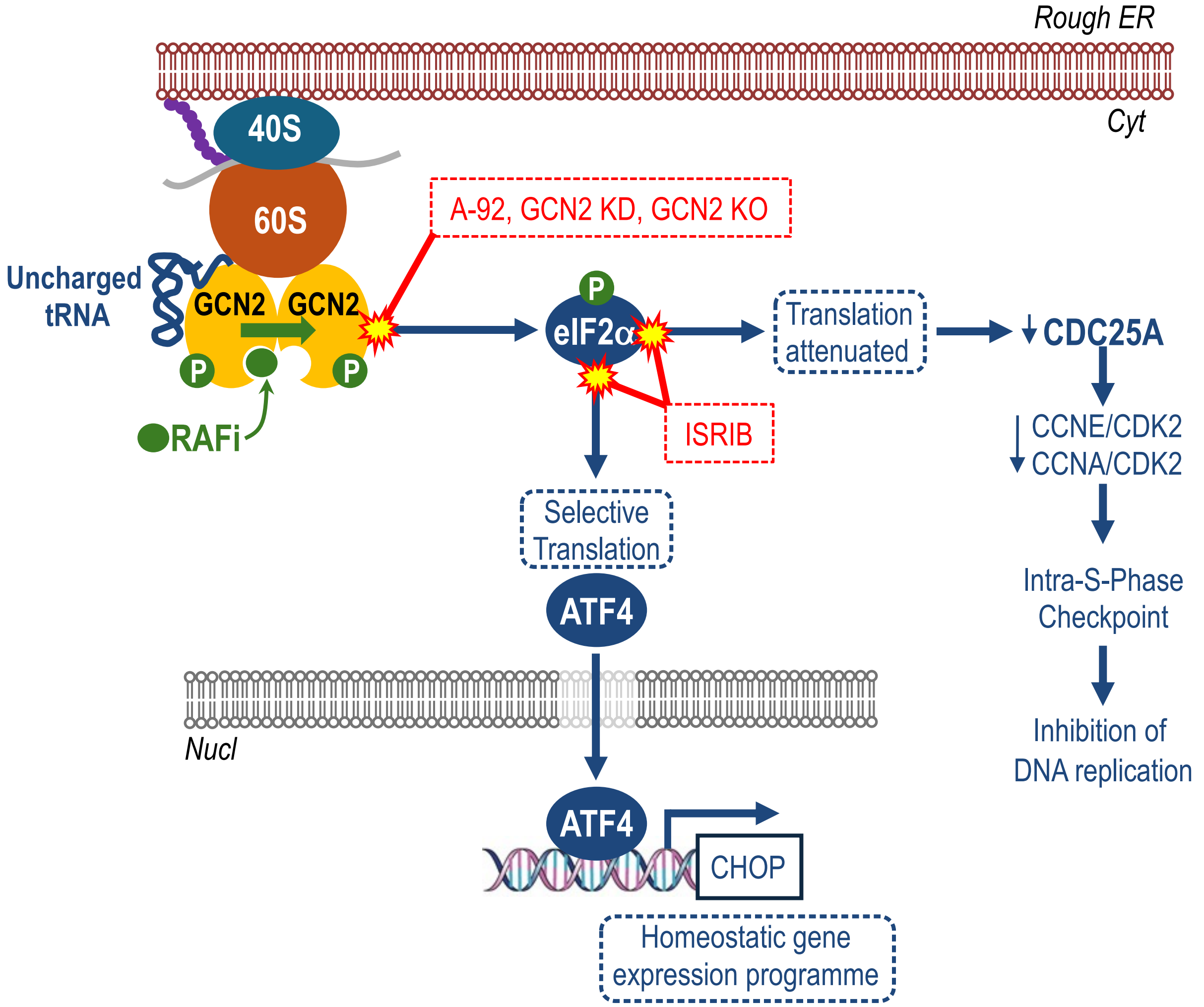

Supplementary Table 1. Full list of reagents and resources used in this study.

REAGENT or RESOURCE	SOURCE	IDENTIFIER
**Antibodies**		
Anti-mouse Alexa Fluor 488 antibody IF 1:500	Thermo Fisher Scientific	Cat# A-11001
Anti-mouse IgG (H+L) (DyLight™ 680 Conjugate) WB 1:15,000	Cell Signaling Technology	Cat# 5470
Anti-mouse IgG (H+L) (DyLight™ 800 4X PEG Conjugate) WB 1:15,000	Cell Signaling Technology	Cat# 5257
Anti-rabbit IgG (H+L) (DyLight™ 680 Conjugate) WB 1:15,000	Cell Signaling Technology	Cat# 5366
Anti-rabbit IgG (H+L) (DyLight™ 800 4X PEG Conjugate) WB 1:15,000	Cell Signaling Technology	Cat# 5151
Anti-mouse IgG (IRDye 800CW conjugate) WB 1:15,000	LI-COR Biosciences	Cat# 926-32210
WB 1:15,000Anti-rabbit IgG (IRDye 800CW conjugate)	LI-COR Biosciences	Cat# 926-32213
αTubulin; mouse monoclonal (clone DM1A) WB 1:10,000	Sigma-Aldrich	Cat# T9026
β-actin; mouse monoclonal (clone AC-15) WB 1:10,000	Sigma-Aldrich	Cat# A5441
ARAF WB 1:500	Cell Signaling Technology	Cat# 4432
ATF4 WB and IF 1:250	Cell Signaling Technology	Cat# 97038
ATF6 WB 1:1000	Cell Signaling Technology	Cat# 5880
BiP WB 1:500	Cell Signaling Technology	Cat# 5889
BRAF IF and WB 1:500	Santa Cruz Biotechnology	Cat# sc-5284
CDC25A; mouse monoclonal (clone F-6) WB 1:200	Santa Cruz Biotechnology	Cat# sc-7389
CHOP (DDIT3) IF and WB 1:250	Cell Signaling Technology	Cat# 2895
Phospho-CHK1 (S354) IF 1:50	Cell Signaling Technology	Cat# 2348
Claspin WB 1:1000	Cell Signaling Technology	Cat# 2800
CRAF WB and IF 1:500	BD	Cat# 610152
Cyclin A; rabbit polyclonal WB 1:500	Santa Cruz Biotechnology	Cat# sc-751
eIF2α WB 1:1000	Cell Signaling Technology	Cat# 9722
Phospho-eIF2α (D9G8) WB 1:1000	Cell Signaling Technology	Cat# 3398
ERK1/2; mouse monoclonal (clone 3A7) WB 1:1000	Cell Signaling Technology	Cat# 9107
ERK1/2; mouse monoclonal (clone L34F12) IF 1:200	Cell Signaling Technology	Cat# 4696,

ERK1/2; rabbit monoclonal (clone 137F5)<br>IF 1:250	Cell Signaling Technology	Cat# 4695
Phospho-ERK1/2 (T202/Y204); rabbit monoclonal (clone D13.14.4E)<br>IF 1:500; WB 1:1000	Cell Signaling Technology	Cat# 4370
Phospho-ERK1/2 (T202/Y204); rabbit polyclonal<br>WB 1:1000	Cell Signaling Technology	Cat# 9101
GCN2<br>WB 1:500	Cell Signaling Technology	Cat# 40457
Phospho-GCN2 (T899) rabbit monoclonal<br>WB 1:250	Abcam<br>Cell Signalling Technology	Cat# ab75836<br>Cat# 94688
HSP90<br>WB 1:10,000	Abcam	Cat# ab13492
GFP rabbit monoclonal<br>WB 1:1000	Cell Signalling Technology	Cat# 2596
Phospho-Histone H2AX (S139)<br>IF 1:1000	Cell Signalling Technology	Cat# 80312
IRE1 (14C10)<br>WB 1:1000	Cell Signalling Technology	Cat# 3294
PERK<br>WB 1:1000	Cell Signaling Technology	Cat# 3192
TRIB3<br>WB 1:1000	Cell Signaling Technology	Cat# 43043
XBP-1<br>IF and WB 1:200	Cell Signaling Technology	Cat# 47143
**Bacterial and Virus Strains**		
NEB 5-alpha competent *E.coli*	New England BioLabs	Cat# C2987
NEB Stable competent *E.coli*	New England BioLabs	Cat# C3040
**Chemicals**		
A92	Med Chem Express	Cat# HY-100877<br>CAS:1448693-69-3
AZ-628	Med Chem Express	Cat# HY-11004<br>CAS:878739-06-1
Dabrafenib	Med Chem Express	Cat# HY-14660<br>CAS:1195765-45-7
Encorafenib	Med Chem Express	Cat# HY-15605<br>CAS:1269440-17-6
Etoposide	Sigma Aldrich (MERCK)	Cat# E1383<br>CAS:33419-42-0
GCN2iB	Med Chem Express	Cat# HY-112654<br>CAS:2183470-12-2
GDC-0879	Med Chem Express	Cat# HY-50864<br>CAS: 905281-76-7
GSK2606414	Sigma Aldrich (MERCK)	Cat# 516535<br>CAS:1337531-89-1
ISRIB	Sigma Aldrich (MERCK)	Cat# SML0843-5MG<br>CAS:1597403-47-8

L-Histidinol dihydrochloride	Sigma Aldrich (MERCK)	Cat# H6647<br>CAS: 1596-64-1
LY-3009120	Med Chem Express	Cat# HY-12558<br>CAS:1454682-72-4
NXP-800	Med Chem Express	Cat# HY-145927<br>CAS: 1693734-80-3
PLX7284	Gift from Plexxicon	
PLX4720	Selleck Chemicals	Cat# S1152;<br>CAS: 918505-84-7
Thapsigargin	Sigma Aldrich (MERCK)	Cat# 586005<br>CAS: 67526-95-8
Trametinib	Selleck Chemicals	Cat# S2673;<br>CAS: 871700-17-3
Tunicamycin	Med Chem Express	Cat# HY-A0098<br>CAS:11089-65-9
Selumetinib (AZD6244)	Provided by AstraZeneca	CAS: 606143-52-6
Vemurafenib	Selleck Chemicals	Cat# S1267;<br>CAS: 918504-65-1
**Experimental Models: Cell Lines**		
Human: HCT116	ATCC	Cat# CCL-247
Human: HT29	ATCC	Cat# HTB-38;
Human: NCI-H338	ATCC	Cat# CRL-5807
Mouse: GCN2 wild-type, GCN2 null, and PERK null mouse embryonic fibroblasts (MEFs)	Ron Lab, Cambridge.	
**Oligonucleotides**	Sigma-Aldrich	N/A
GCN2 amplification<br><br>**GCN2 XHO1_F**<br>gatcgctcgagATGGCTGGGGGCCGTGGGGC<br>**GCN2 XBA1_R**<br>catgtctagaTTAAAATAAGATTCTGTAGTAGTCATCTCTATAG		

Mutagenesis<br><br>**GCN2 802 M to A F**<br>ATACATCCAGGCCGAGTACTGTGAGAAGAGCA C<br>**GCN2 802 M to G_F**<br>ATACATCCAGGGGGAGTACTGTGAGAAGAG<br>**GCN2 802 M to F_F**<br>ATACATCCAGTTCGAGTACTGTGAGAAGAGC<br>**GCN2 802 M to Y_F**<br>ATACATCCAGTACGAGTACTGTGAGAAGAGCAC TTTAC<br>**GCN2 802 M R**<br>AGGTAGTGCACAGCCTCA<br><br>**GCN2 619 K to A_F**<br>CTACGCAGTGGCCCGCATCCCCATC<br>**GCN2 619 K to A_R**<br>CAGCAGCCGTCCAACTTG<br><br>**GCN2 848 D to N_F**<br>GATTCACCGGAATTTGAAGCCTG<br>**GCN2 848 D to N_R**<br>ATTCCTTTCTCATGGATATAAGC		
GCN2 sequencing primers:<br><br>**GCN2 g1 check F**<br>GAGAAAATGCCAATAAGTTGAATAGG<br>**GCN2 g1 check R**<br>CCAAATGAGTAGCAAAAGCCAGAG		
siRNA	Dharmacon	
Non-targeting pool (ON-target plus)		DD-001810-10-2
GCN2 smartpool		L-005314-00-0005
ATF4 smartpool		L-005125-00-0005
ARAF		L-003563-00-0005
BRAF		L-003460-00-0005
CRAF		L-003601-00-0005

Recombinant DNA		
pSpCas9(BB)-2A-GFP genome editing vector	Feng Zhang, Addgene	Cat# 48138
EGFP-C3	Clontech	6081
GST-EIF2AK4	MRC dundee	PPU-DU67392-proteins-703081
hsEIF2AK4_guide1_pSpCas9(BB)-2A-Puro (MP1) (plasmid)	H.Harding, Ron Lab, Cambridge	N/A
hsEIF2AK4_guide2_pSpCas9(BB)-2A-Puro (MP1) (plasmid)	H.Harding, Ron Lab, Cambridge	N/A

Commercial Assays		
Amersham ECL western blotting reagent	Cytiva	Cat# RPN2105
Click-iT EdU Alexa Fluor 647 Flow Cytometry Assay Kit	Thermo Fisher Scientific	Cat# C10424
Click-iT EdU Alexa Fluor 647 HCS Assay Kit	Thermo Fisher Scientific	Cat# C10357
iBLOT2 transfer stacks	Thermo Fisher Scientific	Cat# IB23001
QIAshredder	Qiagen	Cat# 79654
QuickExtract DNA extraction	LGC Biosearch Technologies	Cat# QE0905T
RNeasy mini kit	Qiagen	Cat# 74104
**Deposited or Accessed Data**		
GCN2 Crystal structure	Protein Data Bank	PMID: 15964839
**Software and Algorithms**		
Adobe Photoshop	Adobe Systems Europe Ltd	http://www.adobe.com/uk/products/photoshop.html
FlowJo	FlowJo LLC	https://www.flowjo.com/solutions/flowjo
GraphPad Prism 8	GraphPad Software	https://www.graphpad.com/scientific-software/prism/
Image Studio V5.2	LI-COR Biosciences	https://www.licor.com/bio/products/software/image_studio/
IN Cell Investigator Software	GE Healthcare	https://www.gelifesciences.com/en/us/shop/cell-imaging-and-analysis/high-content-analysis-systems/software/in-cell-investigator-software-p-00344
limma R package	Bioconductor	https://bioconductor.org/packages/release/bioc/html/limma.html

clusterProfiler R package	Bioconductor	https://www.rdocumentation.org/packages/clusterProfiler/versions/3.0.4
