# Supplementary material for: RAF inhibitors activate the integrated stress response by direct activation of GCN2
Source: Nat Commun. 2025 Nov 17;16:10033. doi: 10.1038/s41467-025-65376-w (PMC12623982; doi:10.1038/s41467-025-65376-w)
Supplement: Supplementary file 2 — Reporting Summary [file 41467_2025_65376_MOESM2_ESM.pdf]

nature portfolio

Corresponding author(s): Simon J Cook
Rebecca Gilley

Last updated by author(s): 2024 08 13

Reporting Summary

Nature Portfolio wishes to improve the reproducibility of the work that we publish. This form provides structure for consistency and transparency in reporting. For further information on Nature Portfolio policies, see our Editorial Policies and the Editorial Policy Checklist.

Statistics

For all statistical analyses, confirm that the following items are present in the figure legend, table legend, main text, or Methods section.

n/a	Confirmed	
☐	☒	The exact sample size (*n*) for each experimental group/condition, given as a discrete number and unit of measurement
☒	☐	A statement on whether measurements were taken from distinct samples or whether the same sample was measured repeatedly
☐	☒	The statistical test(s) used AND whether they are one- or two-sided<br>*Only common tests should be described solely by name; describe more complex techniques in the Methods section.*
☒	☐	A description of all covariates tested
☒	☐	A description of any assumptions or corrections, such as tests of normality and adjustment for multiple comparisons
☐	☒	A full description of the statistical parameters including central tendency (e.g. means) or other basic estimates (e.g. regression coefficient) AND variation (e.g. standard deviation) or associated estimates of uncertainty (e.g. confidence intervals)
☐	☒	For null hypothesis testing, the test statistic (e.g. *F*, *t*, *r*) with confidence intervals, effect sizes, degrees of freedom and *P* value noted<br>*Give P values as exact values whenever suitable.*
☒	☐	For Bayesian analysis, information on the choice of priors and Markov chain Monte Carlo settings
☒	☐	For hierarchical and complex designs, identification of the appropriate level for tests and full reporting of outcomes
☒	☐	Estimates of effect sizes (e.g. Cohen's *d*, Pearson's *r*), indicating how they were calculated

Our web collection on statistics for biologists contains articles on many of the points above.

Software and code

Policy information about availability of computer code

Data collection	*Provide a description of all commercial, open source and custom code used to collect the data in this study, specifying the version used OR state that no software was used.*
Data analysis	FlowJo FlowJo LLC https://www.flowjo.com/solutions/flowjo<br>GraphPad Prism 8 GraphPad Software https://www.graphpad.com/scientific-software/prism/<br>Image Studio V5.2 LI-COR Biosciences https://www.licor.com/bio/products/software/image_studio/<br>IN Cell Investigator Software GE Healthcare https://www.gelifesciences.com/en/us/shop/cell-imaging-and-analysis/high-content-analysis-systems/software/in-cell-investigator-software-p-00344<br>limma R package Bioconductor https://bioconductor.org/packages/release/bioc/html/limma.html<br>clusterProfiler R package Bioconductor https://www.rdocumentation.org/packages/clusterProfiler/versions/3.0.4

For manuscripts utilizing custom algorithms or software that are central to the research but not yet described in published literature, software must be made available to editors and reviewers. We strongly encourage code deposition in a community repository (e.g. GitHub). See the Nature Portfolio guidelines for submitting code & software for further information.

Data

Policy information about availability of data

All manuscripts must include a data availability statement. This statement should provide the following information, where applicable:

- Accession codes, unique identifiers, or web links for publicly available datasets
- A description of any restrictions on data availability
- For clinical datasets or third party data, please ensure that the statement adheres to our policy

The RNAseq data are available at Gene Expression Omnibus (https://www.ncbi.nlm.nih.gov/geo/) under accession number GSE271504
All the other data supporting the findings of this study are available within the article and Supplementary Information files. A reporting summary for this article is available in the Supplementary Information.

Research involving human participants, their data, or biological material

Policy information about studies with human participants or human data. See also policy information about sex, gender (identity/presentation), and sexual orientation and race, ethnicity and racism.

Reporting on sex and gender	*Use the terms sex (biological attribute) and gender (shaped by social and cultural circumstances) carefully in order to avoid confusing both terms. Indicate if findings apply to only one sex or gender; describe whether sex and gender were considered in study design; whether sex and/or gender was determined based on self-reporting or assigned and methods used.*<br>*Provide in the source data disaggregated sex and gender data, where this information has been collected, and if consent has been obtained for sharing of individual-level data; provide overall numbers in this Reporting Summary. Please state if this information has not been collected.*<br>*Report sex- and gender-based analyses where performed, justify reasons for lack of sex- and gender-based analysis.*
Reporting on race, ethnicity, or other socially relevant groupings	*Please specify the socially constructed or socially relevant categorization variable(s) used in your manuscript and explain why they were used. Please note that such variables should not be used as proxies for other socially constructed/relevant variables (for example, race or ethnicity should not be used as a proxy for socioeconomic status).*<br>*Provide clear definitions of the relevant terms used, how they were provided (by the participants/respondents, the researchers, or third parties), and the method(s) used to classify people into the different categories (e.g. self-report, census or administrative data, social media data, etc.)*<br>*Please provide details about how you controlled for confounding variables in your analyses.*
Population characteristics	*Describe the covariate-relevant population characteristics of the human research participants (e.g. age, genotypic information, past and current diagnosis and treatment categories). If you filled out the behavioural & social sciences study design questions and have nothing to add here, write "See above."*
Recruitment	*Describe how participants were recruited. Outline any potential self-selection bias or other biases that may be present and how these are likely to impact results.*
Ethics oversight	*Identify the organization(s) that approved the study protocol.*

Note that full information on the approval of the study protocol must also be provided in the manuscript.

Field-specific reporting

Please select the one below that is the best fit for your research. If you are not sure, read the appropriate sections before making your selection.

☒ Life sciences ☐ Behavioural & social sciences ☐ Ecological, evolutionary & environmental sciences

For a reference copy of the document with all sections, see nature.com/documents/nr-reporting-summary-flat.pdf

Life sciences study design

All studies must disclose on these points even when the disclosure is negative.

Sample size	For high content image analysis 2000-15000 cells per condition were used.<br>In FLOW cytometry a minimum of 10,000 cells were counted.<br>These sample sizes were based on previous studies by our lab and others in the field which have generated statistically robust data<br>Reproducibility of in vitro experiments was determined by full repetition of experiments, typically on three separate occasions, and performing statistical tests where appropriate.
Data exclusions	No data was excluded
Replication	All experiments were repeated at least 3 times and confirmed the same results by independent investigators. Results were confirmed in several different cell lines.
Randomization	Drug treatment groups were all performed on the same cell lines. The outcome of these treatments was unknown but had untreated controls.

Blinding	Investigators were not blinded. However, virtually all experiments were repeated by up to three independent investigators using complemetary orthogonal methods. Experiements were using drug treatments so blinding is not relevant or possible.

Reporting for specific materials, systems and methods

We require information from authors about some types of materials, experimental systems and methods used in many studies. Here, indicate whether each material, system or method listed is relevant to your study. If you are not sure if a list item applies to your research, read the appropriate section before selecting a response.

Materials & experimental systems

n/a	Involved in the study
☐	☒ Antibodies
☐	☒ Eukaryotic cell lines
☐	☐ Palaeontology and archaeology
☐	☐ Animals and other organisms
☐	☐ Clinical data
☐	☐ Dual use research of concern
☐	☐ Plants

Methods

n/a	Involved in the study
☐	☐ ChIP-seq
☐	☒ Flow cytometry
☐	☐ MRI-based neuroimaging

Antibodies

Antibodies used

Anti-mouse Alexa Fluor 488 antibody Thermo Fisher Scientific Cat# A-11001 IF 1:500
Anti-mouse IgG (H+L) (DyLight™ 680 Conjugate) Cell Signaling Technology Cat# 5470 WB 1:15,000
Anti-mouse IgG (H+L) (DyLight™ 800 4X PEG Conjugate) Cell Signaling Technology Cat# 5257 WB 1:15,000
Anti-rabbit IgG (H+L) (DyLight™ 680 Conjugate) Cell Signaling Technology Cat# 5366 WB 1:15,000
Anti-rabbit IgG (H+L) (DyLight™ 800 4X PEG Conjugate) Cell Signaling Technology Cat# 5151
Anti-mouse IgG (IRDye 800CW conjugate) LI-COR Biosciences Cat# 926-32210 WB 1:15,000
Anti-rabbit IgG (IRDye 800CW conjugate) LI-COR Biosciences Cat# 926-32213 WB 1:15,000
αTubulin; mouse monoclonal (clone DM1A) Sigma-Aldrich Cat# T9026 WB 1:10,000
β-actin; mouse monoclonal (clone AC-15) Sigma-Aldrich Cat# A5441 WB 1;10,000
ARAF Cell Signaling Technology Cat# 4432 WB 1:500
ATF4 Cell Signaling Technology Cat# 97038 WB and IF 1:250
ATF6 Cell Signaling Technology Cat# 5880 WB 1:1000
BiP Cell Signaling Technology Cat# 5889 WB 1:500
BRAF Santa Cruz Biotechnology Cat# sc-5284 IF and WB 1:500
CDC25A; mouse monoclonal (clone F-6) Santa Cruz Biotechnology Cat# sc-7389 WB 1:200
CHOP (DDIT3) Cell Signaling Technology Cat# 2895 IF and WB 1:250
Phospho-CHK1 (S354) Cell Signaling Technology Cat# 2348 IF 1:50
Claspin Cell Signaling Technology Cat# 2800 WB 1:1000
CRAF BD Cat# 610152 WB and IF 1:500
Cyclin A; rabbit polyclonal Santa Cruz Biotechnology Cat# sc-751 WB 1:500
eIF2α Cell Signaling Technology Cat# 9722 WB 1:1000
Phospho-eIF2α (D9G8) Cell Signaling Technology Cat# 3398 WB 1:1000
ERK1/2; mouse monoclonal (clone 3A7) Cell Signaling Technology Cat# 9107 WB 1:1000
ERK1/2; mouse monoclonal (clone L34F12) Cell Signaling Technology Cat# 4696 IF 1:200
ERK1/2; rabbit monoclonal (clone 137F5) Cell Signaling Technology Cat# 4695 IF 1:250
Phospho-ERK1/2 (T202/Y204); rabbit monoclonal (clone D13.14.4E) Cell Signaling Technology Cat# 4370
Phospho-ERK1/2 (T202/Y204); rabbit polyclonal Cell Signaling Technology Cat# 9101 IF 1:500; WB 1:1000
GCN2 Cell Signaling Technology Cat# 40457 WB 1:500
Phospho-GCN2 (T899) rabbit monoclonal Abcam Cat# ab75836 and Cell Signalling Technology Cat# 94688
HSP90 Abcam Cat# ab13492 WB 1:250
GFP rabbit monoclonal Cell Signalling Technology Cat# 2596 WB 1:10,000
Phospho-Histone H2AX (S139) Cell Signalling Technology Cat# 80312 IF 1:1000
IRE1 (14C10) Cell Signalling Technology Cat# 3294 WB 1:1000
PERK Cell Signaling Technology Cat# 3192 WB 1:1000
TRIB3 Cell Signaling Technology Cat# 43043 WB 1:1000
XBP-1 Cell Signaling Technology Cat# 4714 IF and WB 1:200

Validation

all antibodies have been validated by manufacturers, refer to catolgue numbers.

The following antibodies were validated in our hands through the use of siRNA or by testing on relevant gene knockout cell lines:-
ARAF Cell Signaling Technology Cat# 4432 suppl fig 8
ATF4 Cell Signaling Technology Cat# 97038 suppl. fig 6

BRAF Santa Cruz Biotechnology Cat# sc-5284 suppl fig 8
CHOP (DDIT3) Cell Signaling Technology Cat# 2895 fig 5d
CRAF BD Cat# 610152 suppl fig 8
Phospho-eIF2α (D9G8) Cell Signaling Technology Cat# 3398 fig 5d
GCN2 Cell Signaling Technology Cat# 40457 fig 5b
Phospho-GCN2 (T899) rabbit monoclonal Abcam Cat# ab75836 and Cell Signalling Technology Cat# 94688 fig 5b
PERK Cell Signaling Technology Cat# 3192

Many antibodies were validated by their previous use by our own lab and other leaders in the field and by their ability to detect the relevant protein under very specific - and protein-defining - conditions (stimulation, inhibition) by western blot or IF.
Two examples underline this principle.
The Phospho-ERK1/2 (T202/Y204); rabbit monoclonal (clone D13.14.4E) and Cell Signaling Technology Cat# 4370 only detected ERK1/2 (at 44kDa and 42 kDa) when they had undergone activation phosphorylation in response to paradoxical RAF activation.
The BiP Cell Signaling Technology Cat# 5889 antibody detected the two BiP proteins at 70-78kDa that were expressed very strongly in response to ER stress stimuli.

The antibodies validated in this way were:-
ATF4 Cell Signaling Technology Cat# 97038
ATF6 Cell Signaling Technology Cat# 5880
BiP Cell Signaling Technology Cat# 5889
CDC25A; mouse monoclonal (clone F-6) Santa Cruz Biotechnology Cat# sc-7389
CHOP (DDIT3) Cell Signaling Technology Cat# 2895
Phospho-CHK1 (S354) Cell Signaling Technology Cat# 2348
Claspin Cell Signaling Technology Cat# 2800
Phospho-eIF2α (D9G8) Cell Signaling Technology Cat# 3398
Phospho-ERK1/2 (T202/Y204); rabbit monoclonal (clone D13.14.4E) Cell Signaling Technology Cat# 4370
Phospho-ERK1/2 (T202/Y204); rabbit polyclonal Cell Signaling Technology Cat# 9101
GFP rabbit monoclonal Cell Signalling Technology Cat# 2596
Phospho-Histone H2AX (S139) Cell Signalling Technology Cat# 80312
IRE1 (14C10) Cell Signalling Technology Cat# 3294
PERK Cell Signaling Technology Cat# 3192
TRIB3 Cell Signaling Technology Cat# 43043
XBP-1 Cell Signaling Technology Cat# 47143

The remaining antibodies were validated in the manufacturers data sheets and have become standard reagents used by many laboratories. These include:-
Anti-mouse Alexa Fluor 488 antibody Thermo Fisher Scientific Cat# A-11001
Anti-mouse IgG (H+L) (DyLight™ 680 Conjugate) Cell Signaling Technology Cat# 5470
Anti-mouse IgG (H+L) (DyLight™ 800 4X PEG Conjugate) Cell Signaling Technology Cat# 5257
Anti-rabbit IgG (H+L) (DyLight™ 680 Conjugate) Cell Signaling Technology Cat# 5366
Anti-rabbit IgG (H+L) (DyLight™ 800 4X PEG Conjugate) Cell Signaling Technology Cat# 5151
Anti-mouse IgG (IRDye 800CW conjugate) LI-COR Biosciences Cat# 926-32210
Anti-rabbit IgG (IRDye 800CW conjugate) LI-COR Biosciences Cat# 926-32213
αTubulin; mouse monoclonal (clone DM1A) Sigma-Aldrich Cat# T9026
β-actin; mouse monoclonal (clone AC-15) Sigma-Aldrich Cat# A5441
ERK1/2; mouse monoclonal (clone 3A7) Cell Signaling Technology Cat# 9107
ERK1/2; mouse monoclonal (clone L34F12) Cell Signaling Technology Cat# 4696,
ERK1/2; rabbit monoclonal (clone 137F5) Cell Signaling Technology Cat# 4695
HSP90 Abcam Cat# ab13492

Eukaryotic cell lines

Policy information about cell lines and Sex and Gender in Research

Cell line source(s)	Human: HCT116 ATCC Cat# CCL-247 (adult male)<br><br>Human: HT29 ATCC Cat# HTB-38; (adult female)<br><br>Human: NCI-H338 ATCC Cat# CRL-5807 (Adult male)<br><br>Mouse: GCN2 wild-type, GCN2 null, and PERK null mouse embryonic fibroblasts (MEFs) Ron Lab, Cambridge.
Authentication	All cell lines were authenticated by Short Tandem Repeat (STR) profiling
Mycoplasma contamination	All cell lines tested negative for mycoplasma

Commonly misidentified lines (See ICLAC register)	None

Palaeontology and Archaeology

Specimen provenance	*Provide provenance information for specimens and describe permits that were obtained for the work (including the name of the issuing authority, the date of issue, and any identifying information). Permits should encompass collection and, where applicable, export.*
Specimen deposition	*Indicate where the specimens have been deposited to permit free access by other researchers.*
Dating methods	*If new dates are provided, describe how they were obtained (e.g. collection, storage, sample pretreatment and measurement), where they were obtained (i.e. lab name), the calibration program and the protocol for quality assurance OR state that no new dates are provided.*

☐ Tick this box to confirm that the raw and calibrated dates are available in the paper or in Supplementary Information.

Ethics oversight	*Identify the organization(s) that approved or provided guidance on the study protocol, OR state that no ethical approval or guidance was required and explain why not.*

Note that full information on the approval of the study protocol must also be provided in the manuscript.

Animals and other research organisms

Policy information about studies involving animals; ARRIVE guidelines recommended for reporting animal research, and Sex and Gender in Research

Laboratory animals	*For laboratory animals, report species, strain and age OR state that the study did not involve laboratory animals.*
Wild animals	*Provide details on animals observed in or captured in the field; report species and age where possible. Describe how animals were caught and transported and what happened to captive animals after the study (if killed, explain why and describe method; if released, say where and when) OR state that the study did not involve wild animals.*
Reporting on sex	*Indicate if findings apply to only one sex; describe whether sex was considered in study design, methods used for assigning sex. Provide data disaggregated for sex where this information has been collected in the source data as appropriate; provide overall numbers in this Reporting Summary. Please state if this information has not been collected. Report sex-based analyses where performed, justify reasons for lack of sex-based analysis.*
Field-collected samples	*For laboratory work with field-collected samples, describe all relevant parameters such as housing, maintenance, temperature, photoperiod and end-of-experiment protocol OR state that the study did not involve samples collected from the field.*
Ethics oversight	*Identify the organization(s) that approved or provided guidance on the study protocol, OR state that no ethical approval or guidance was required and explain why not.*

Note that full information on the approval of the study protocol must also be provided in the manuscript.

Clinical data

Policy information about clinical studies

All manuscripts should comply with the ICMJE guidelines for publication of clinical research and a completed CONSORT checklist must be included with all submissions.

Clinical trial registration	*Provide the trial registration number from ClinicalTrials.gov or an equivalent agency.*
Study protocol	*Note where the full trial protocol can be accessed OR if not available, explain why.*
Data collection	*Describe the settings and locales of data collection, noting the time periods of recruitment and data collection.*
Outcomes	*Describe how you pre-defined primary and secondary outcome measures and how you assessed these measures.*

Dual use research of concern

Policy information about dual use research of concern

Hazards

Could the accidental, deliberate or reckless misuse of agents or technologies generated in the work, or the application of information presented in the manuscript, pose a threat to:

No	Yes	
☒	☐	Public health
☒	☐	National security
☒	☐	Crops and/or livestock
☒	☐	Ecosystems
☒	☐	Any other significant area

Experiments of concern

Does the work involve any of these experiments of concern:

No	Yes	
☒	☐	Demonstrate how to render a vaccine ineffective
☒	☐	Confer resistance to therapeutically useful antibiotics or antiviral agents
☒	☐	Enhance the virulence of a pathogen or render a nonpathogen virulent
☒	☐	Increase transmissibility of a pathogen
☒	☐	Alter the host range of a pathogen
☒	☐	Enable evasion of diagnostic/detection modalities
☒	☐	Enable the weaponization of a biological agent or toxin
☒	☐	Any other potentially harmful combination of experiments and agents

Plants

Seed stocks	*Report on the source of all seed stocks or other plant material used. If applicable, state the seed stock centre and catalogue number. If plant specimens were collected from the field, describe the collection location, date and sampling procedures.*
Novel plant genotypes	*Describe the methods by which all novel plant genotypes were produced. This includes those generated by transgenic approaches, gene editing, chemical/radiation-based mutagenesis and hybridization. For transgenic lines, describe the transformation method, the number of independent lines analyzed and the generation upon which experiments were performed. For gene-edited lines, describe the editor used, the endogenous sequence targeted for editing, the targeting guide RNA sequence (if applicable) and how the editor was applied.*
Authentication	*Describe any authentication procedures for each seed stock used or novel genotype generated. Describe any experiments used to assess the effect of a mutation and, where applicable, how potential secondary effects (e.g. second site T-DNA insertions, mosiacism, off-target gene editing) were examined.*

ChIP-seq

Data deposition

☐ Confirm that both raw and final processed data have been deposited in a public database such as GEO.

☐ Confirm that you have deposited or provided access to graph files (e.g. BED files) for the called peaks.

Data access links<br>*May remain private before publication.*	*For "Initial submission" or "Revised version" documents, provide reviewer access links. For your "Final submission" document, provide a link to the deposited data.*
Files in database submission	*Provide a list of all files available in the database submission.*
Genome browser session<br>(e.g. UCSC)	*Provide a link to an anonymized genome browser session for "Initial submission" and "Revised version" documents only, to enable peer review. Write "no longer applicable" for "Final submission" documents.*

Methodology

Replicates	*Describe the experimental replicates, specifying number, type and replicate agreement.*
Sequencing depth	*Describe the sequencing depth for each experiment, providing the total number of reads, uniquely mapped reads, length of reads and whether they were paired- or single-end.*
Antibodies	*Describe the antibodies used for the ChIP-seq experiments; as applicable, provide supplier name, catalog number, clone name, and lot number.*
Peak calling parameters	*Specify the command line program and parameters used for read mapping and peak calling, including the ChIP, control and index files used.*
Data quality	*Describe the methods used to ensure data quality in full detail, including how many peaks are at FDR 5% and above 5-fold enrichment.*

Software

Describe the software used to collect and analyze the ChIP-seq data. For custom code that has been deposited into a community repository, provide accession details.

Flow Cytometry

Plots

Confirm that:

- [x] The axis labels state the marker and fluorochrome used (e.g. CD4-FITC).
- [x] The axis scales are clearly visible. Include numbers along axes only for bottom left plot of group (a 'group' is an analysis of identical markers).
- [x] All plots are contour plots with outliers or pseudocolor plots.
- [ ] A numerical value for number of cells or percentage (with statistics) is provided.

Methodology

Sample preparation

Cells were harvested by trypsinisation and fixed with 4% paraformaldehyde/PBS for 10 min at room temperature. EdU was detected following the manufacturer's instructions, and cells were resuspended in 1µg/mL DAPI/PBS (Sigma-Aldrich, Dorset, UK).

Instrument

FACS LSRII (BD Biosciences, Oxford, UK),

Software

For acquistion BD FACSDiva software was used and Flow JO for analysis

Cell population abundance

Describe the abundance of the relevant cell populations within post-sort fractions, providing details on the purity of the samples and how it was determined.

Gating strategy

SSCA v FSCA to get a single cell population and eliminate debris, followed by both FSC H v FSC A and SSC H v SSC A gates to eliminates doublets. Edu (click it 647) and DAPI stained and gated for cell cycle phases.

- [ ] Tick this box to confirm that a figure exemplifying the gating strategy is provided in the Supplementary Information.

Magnetic resonance imaging

Experimental design

Design type

Indicate task or resting state; event-related or block design.

Design specifications

Specify the number of blocks, trials or experimental units per session and/or subject, and specify the length of each trial or block (if trials are blocked) and interval between trials.

Behavioral performance measures

State number and/or type of variables recorded (e.g. correct button press, response time) and what statistics were used to establish that the subjects were performing the task as expected (e.g. mean, range, and/or standard deviation across subjects).

Acquisition

Imaging type(s)

Specify: functional, structural, diffusion, perfusion.

Field strength

Specify in Tesla

Sequence & imaging parameters

Specify the pulse sequence type (gradient echo, spin echo, etc.), imaging type (EPI, spiral, etc.), field of view, matrix size, slice thickness, orientation and TE/TR/flip angle.

Area of acquisition

State whether a whole brain scan was used OR define the area of acquisition, describing how the region was determined.

Diffusion MRI ☐ Used ☐ Not used

Preprocessing

Preprocessing software

Provide detail on software version and revision number and on specific parameters (model/functions, brain extraction, segmentation, smoothing kernel size, etc.).

Normalization

If data were normalized/standardized, describe the approach(es): specify linear or non-linear and define image types used for transformation OR indicate that data were not normalized and explain rationale for lack of normalization.

Normalization template

Describe the template used for normalization/transformation, specifying subject space or group standardized space (e.g. original Talairach, MNI305, ICBM152) OR indicate that the data were not normalized.

Noise and artifact removal

Describe your procedure(s) for artifact and structured noise removal, specifying motion parameters, tissue signals and physiological signals (heart rate, respiration).

Volume censoring

Define your software and/or method and criteria for volume censoring, and state the extent of such censoring.

Statistical modeling & inference

Model type and settings

Specify type (mass univariate, multivariate, RSA, predictive, etc.) and describe essential details of the model at the first and second levels (e.g. fixed, random or mixed effects; drift or auto-correlation).

Effect(s) tested

Define precise effect in terms of the task or stimulus conditions instead of psychological concepts and indicate whether ANOVA or factorial designs were used.

Specify type of analysis: ☐ Whole brain ☐ ROI-based ☐ Both

Statistic type for inference

(See Eklund et al. 2016)

Specify voxel-wise or cluster-wise and report all relevant parameters for cluster-wise methods.

Correction

Describe the type of correction and how it is obtained for multiple comparisons (e.g. FWE, FDR, permutation or Monte Carlo).

Models & analysis

n/a	Involved in the study
☐	☐ Functional and/or effective connectivity
☐	☐ Graph analysis
☐	☐ Multivariate modeling or predictive analysis

Functional and/or effective connectivity

Report the measures of dependence used and the model details (e.g. Pearson correlation, partial correlation, mutual information).

Graph analysis

Report the dependent variable and connectivity measure, specifying weighted graph or binarized graph, subject- or group-level, and the global and/or node summaries used (e.g. clustering coefficient, efficiency, etc.).

Multivariate modeling and predictive analysis

Specify independent variables, features extraction and dimension reduction, model, training and evaluation metrics.
